# Low temperatures impact on the expression of growth genes and miRNAs in Nile tilapia (*Oreochromis niloticus*)

**DOI:** 10.1007/s10695-026-01679-z

**Published:** 2026-04-10

**Authors:** Eduardo N. Dellagostin, Natiéli M. Gonçalves, Eduardo B. Blödorn, Amanda W. S. Martins, Guilherme N. L. Rattmann, Kaylane P. Vasconcelos, Gilberto L. Collares, Tony L. R. Silveira, Mariana H. Remião, Vinicius F. Campos

**Affiliations:** 1 Laboratório de Genômica Estrutural, Programa de Pós-Graduação em Biotecnologia, Centro de Desenvolvimento Tecnológico, Universidade Federal de Pelotas, Campus Universitário Capão do Leão s/nº - Prédio 20, Jardim América, Capão do Leão, CEP: 96.010-900, Pelotas, RS Brasil; 2 Núcleo de Ensino, Pesquisa e Extensão em Hidrometria e Sedimentologia para o Manejo de Bacias Hidrográficas (Hidrosedi), Centro de Desenvolvimento Tecnológico, Universidade Federal de Pelotas, Pelotas, RS Brasil

**Keywords:** Cold stress, MicroRNA, Growth regulation, Nile tilapia, Gene expression

## Abstract

Nile tilapia (*Oreochromis niloticus*) is one of the most important aquaculture species worldwide, but its sensitivity to low temperatures limits production, particularly in subtropical and temperate regions. Since somatic growth in fish is largely regulated by the growth hormone/insulin-like growth factor (GH/IGF) axis, and microRNAs contribute to the post-transcriptional regulation of growth, metabolism, and stress responses, this study investigated the molecular effects of chronic cold exposure in Nile tilapia by analysing growth-related genes and five candidate microRNAs (*miR-192, miR-206, miR-133, miR-125b*, and *miR-30b*) in the brain, liver, and hindgut after 28 days at 15 °C. Cold exposure induced marked tissue-specific transcriptional changes. In the liver and hindgut, *gh1*, *igf-1*, and *igf-2* were generally upregulated, whereas in the brain *gh1* expression increased while *ghra*, *igf-1*, and *igf-2* were reduced. MicroRNA expression also differed among tissues: in the brain, *miR-192* and *miR-206* were upregulated and *miR-125b* was downregulated; in the liver, *miR-133* and *miR-206* increased whereas *miR-192* decreased; and in the hindgut, *miR-192*, *miR-125b*, and *miR-206* were downregulated. The concurrent upregulation of *miR-206* and *igf-1* in the liver suggests a complex and tissue-dependent regulatory relationship under cold stress. Overall, these findings demonstrate that chronic low-temperature exposure modulates both the GH/IGF axis and miRNA expression in a tissue-specific manner, highlighting integrated transcriptional and post-transcriptional mechanisms involved in cold adaptation. This study provides new insight into the molecular basis of thermal resilience in Nile tilapia and supports the identification of candidate biomarkers for cold tolerance in aquaculture.

**Graphical Abstract:**

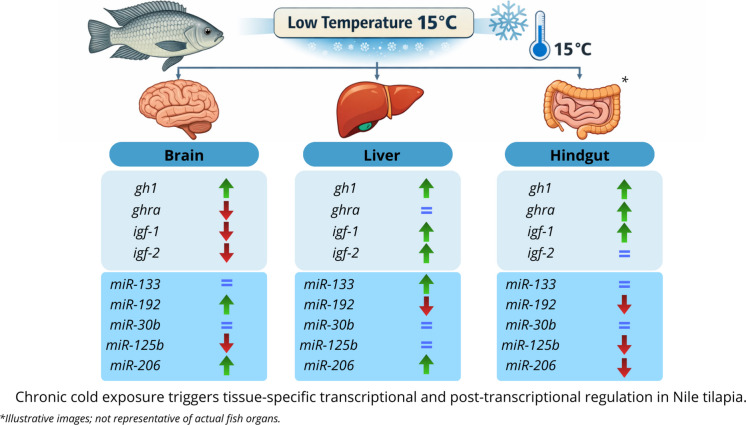

**Supplementary Information:**

The online version contains supplementary material available at 10.1007/s10695-026-01679-z.

## Introduction

The Nile tilapia (*Oreochromis niloticus*) is one of the most widely produced fish species globally, with Brazil ranking among the top producers alongside China, Indonesia, Egypt, the Philippines, and Thailand (Pedrini et al. 2024; Silva and Barros 2020). The species is valued for its rapid growth, high reproductive capacity, and economic relevance to aquaculture (FAO 2024). According to the Food and Agriculture Organization (FAO), global tilapia production surpassed 7 million metric tons in 2024, consolidating its status as one of the most profitable farmed species worldwide, with production expected to continue expanding in the coming years (FAO 2024). In Brazil, inland tilapia farming increased by 5% in 2023, with projections indicating a 15% rise in 2024. Most production occurs in subtropical regions, particularly in the South and Southeast of the country (Pedrini et al. 2024; Valenti et al. 2021). However, tilapia growth in subtropical and Mediterranean-type climates is strongly influenced by abiotic factors, especially prolonged cold winter periods, due to the species’ marked sensitivity to low temperatures.

Low temperature is therefore a biologically and economically relevant stressor for Nile tilapia. The optimal rearing temperature for the species is close to 28 °C, at which growth and feed conversion are maximized. At 15 °C, feeding is markedly reduced or completely interrupted, growth performance declines substantially, and temperatures approaching 10 °C may cause high mortality (Dellagostin et al. 2022). As ectothermic organisms, tilapia depend on environmental temperature to regulate metabolic rate, endocrine signalling, feeding activity, immune competence, and reproductive function (Donaldson et al. 2008; Mininni et al. 2014). Exposure to cold can impair immune responses (Abram et al. 2017), reduce growth (Abd El-Hack et al. 2022), alter gonadal development (Melo et al. 2016), and increase oxidative stress (Qiang et al. 2018; Zhou et al. 2019). For these reasons, low-temperature challenge provides a relevant experimental model for investigating the molecular mechanisms that underlie thermal adaptation and cold tolerance in this species.

Among the pathways most closely associated with somatic growth and metabolic regulation in fish is the growth hormone/insulin-like growth factor (GH/IGF) axis. Growth hormone (GH), secreted by the pituitary, acts through the growth hormone receptor (GHR) and stimulates downstream mediators such as insulin-like growth factor 1 (IGF-1) and insulin-like growth factor 2 (IGF-2), especially in the liver, thereby coordinating nutrient partitioning, protein accretion, and tissue growth (Bertucci et al. 2019). In teleosts, this axis also interfaces with osmoregulation, reproduction, and the physiological response to environmental stressors (Biga and Meyer 2009; Canosa and Bertucci 2023). Because cold exposure suppresses feed intake and alters energy balance, it is expected to affect the GH/IGF axis and the transcriptional programs associated with growth maintenance (Escobar-Aguirre et al. 2020). In addition to nutritional cues, post-transcriptional regulation mediated by microRNAs (miRNAs) also modulates tilapia growth by targeting mRNAs involved in the GH/IGF axis (Huang et al. 2012; Yan et al. 2012).

In parallel with endocrine regulation, microRNAs (miRNAs) are important post-transcriptional modulators of growth, metabolic control, and stress responsiveness. MiRNAs are small non-coding RNAs of approximately 22 nucleotides that bind to complementary sequences in the 3′ untranslated region of target (UTR) mRNAs, promoting mRNA degradation or translational repression (Cao et al. 2023; Herkenhoff et al. 2018). In teleost fish, miRNAs play essential roles in embryogenesis, reproduction, tissue regeneration, and responses to environmental stressors such as salinity, temperature, and oxygen fluctuations (Bizuayehu and Babiak 2014; Cao et al. 2023). In zebrafish (*Danio rerio*), for example, miRNAs have been extensively studied for their involvement in regulating gene expression across developmental and physiological processes (Bhattacharya et al. 2017).

The selection of the microRNAs analyzed in this study was based on prior evidence linking these molecules to growth regulation, metabolic control, and stress responsiveness in teleost fish and other vertebrate models. MiR-206 and miR-133 are classically described as myomiRs and have been implicated in the regulation of the GH/IGF axis, particularly through modulation of igf-1 expression and IGF signaling pathways (Mishima et al. 2009; Huang et al. 2012). Notably, miR-206 has been experimentally validated as a post-transcriptional regulator of igf-1 in Nile tilapia, highlighting its relevance to growth control under environmentally challenging conditions (Yan et al. 2012). MiR-192 has been associated with metabolic regulation and insulin-related signaling and has been reported to target components of the IGF-binding protein network, suggesting a potential indirect role in growth modulation (Li et al. 2021). MiR-125b is involved in the regulation of the PI3K/Akt pathway and cellular stress responses, processes tightly linked to growth suppression and energy conservation during adverse environmental conditions (Fu et al. 2020). Additionally, miR-30b has been proposed as a candidate biomarker of thermal stress in teleosts, particularly under acute cold exposure, although its role during chronic cold stress remains poorly understood (Blödorn et al. 2021). Despite these individual associations, the coordinated interaction between these miRNAs and the GH/IGF growth axis during chronic low-temperature exposure has not been systematically investigated in a tissue-specific manner in Nile tilapia.

Low temperatures have substantial effects on fish physiological processes, yet their influence on post-transcriptional regulation remains poorly understood. Therefore, this study aims to elucidate the impact of chronic low-temperature exposure on the expression of growth-related genes and regulatory miRNAs in Nile tilapia.

## Materials and methods

### Animal husbandry and experimental design

All animal procedures were conducted in compliance with ethical standards and were approved by the Ethics Committee on Animal Experimentation of the Federal University of Pelotas (Protocol No. 23110.014105/2020–56). Nile tilapia (*Oreochromis niloticus*) were obtained from a commercial supplier and maintained at the Laboratory of Fish Hatchery, located at the Chasqueiro Dam Fish Farming Unit (Arroio Grande, Brazil; 32°14′15″S, 53°05′13″W). Fish were reared until they reached an average weight of 120 ± 20 g prior to the experimental period.

During a four-week acclimation period, fish were held in a freshwater recirculating system composed of 1,000 L tanks with a working volume of 650 L. A total of 60 fish were randomly assigned to two experimental groups (control and cold-exposed), with each group consisting of three replicate tanks containing ten fish per tank. Throughout acclimation, fish were fed to satiety three times daily using a commercial diet containing 38% crude protein (Supra, Alisul, Brazil). Water temperature was kept at 24 ± 1.5 °C under natural photoperiod conditions, and the system water was partially renewed every 48 h with temperature-adjusted freshwater.

Following acclimation, the control group remained at 24 °C, whereas the cold-exposed group was subjected to gradual cooling at a rate of 0.5 °C per day until the water reached 15 °C. This target temperature reflects typical winter conditions in Southern Brazil according to the Brazilian National Institute of Meteorology (INMET, 2024). Once the target temperature was reached, fish remained under their respective thermal conditions for 28 days in the same recirculating system and at the same stocking density used during acclimation. Water temperature and physicochemical parameters were monitored daily throughout the experimental period. Fish in the control group continued to receive the same commercial diet to satiety three times daily. During cooling, the cold-exposed group was offered the same diet, but feed intake progressively decreased as water temperature declined, consistent with the known reduction in feeding at low temperatures in this species (Dellagostin et al. 2022). To minimize variation associated with recent feed intake, all fish were fasted for 12 h prior to sampling.

At the end of the 28-day cold exposure period, fish were gently netted and anesthetized in 10 L of water containing 400 mg L⁻^1^ tricaine methanesulfonate (MS-222), following the protocol described by Dellagostin et al. (2022), until complete loss of reflexes. Fish were then euthanized by spinal severance for tissue collection. Each individual fish was considered an independent biological replicate, and six fish per group were used for molecular analyses (*n* = 6 per group; *n* = 12 total). Brain, liver, and hindgut samples were immediately snap-frozen in liquid nitrogen and stored at − 80 °C for subsequent RNA extraction. Tissues were collected and processed individually, with no pooling of samples at any stage. Thus, the sample size indicated in the figures refers to the total number of biological replicates analyzed across both experimental groups. The remaining fish described in the experimental design were not included in the molecular analyses.

### RNA extraction and cDNA synthesis

Total RNA was isolated from collected tissues using TRIzol Reagent (Thermo Fisher Scientific, USA), following the protocol provided by the manufacturer. To prevent contamination with genomic DNA, samples were treated with DNase I using the DNase-Free Kit (Ambion, USA). RNA quantity and purity were evaluated using a NanoVue Plus spectrophotometer (GE Healthcare Life Sciences, USA), and all samples were stored at −80 °C until further use.

Complementary DNA (cDNA) was synthesized from 500 ng of total RNA using the High-Capacity cDNA Reverse Transcription Kit (Applied Biosystems, USA), according to the manufacturer’s recommendations. For microRNA analysis, reverse transcription was performed using the stem-loop primer methodology, with specific primers designed for each target miRNA in combination with the same cDNA synthesis kit (Chen et al. 2005). Primer sequences used for cDNA synthesis are listed in Table 1.

**Table 1:** List of primers for specific miRNA cDNA synthesis and real-time quantitative PCR (q-PCR) analysis in this study

miRNA name	Primer sequences (5’ → 3’)	Reference
miR-30b	SL: GTCGTATCCAGTGCAGGGTCCGAGGTATTCGCACTGGATACGACAGCT F: GGCGTGTAAACATCCTCAACTC R: GTGCAGGGTCCGAGGT	(Blödorn et al. 2024)
miR-125b	SL: GTCGTATCCAGTGCAGGGTCCGAGGTATTCGCACTGGATACGACTCACAAGT F: GCATCCTCCCTGAGACCCTA R: CAGTGCAGGGTCCGAGGTAT	(Yu et al. 2020)
miR-133	SL: GTTGGCTCTGGTGCAGGGTCCGAGGTATTCGCACCAGAGCCAACCAGCTG F: GCGGTTTGGTCCCCTTCAAC R: GTGCAGGGTCCGAGGT	(Huang et al. 2012)
miR-192	SL: GTTGGCTCTGGTGCAGGGTCCGAGGTATTCGCACCAGAGCCAACGGCTGT F: CGGCGGATGACCTATGAATTG R: GTGCAGGGTCCGAGGT	(Huang et al. 2012)
miR-206	SL: GTTGGCTCTGGTGCAGGGTCCGAGGTATTCGCACCAGAGCCAACCCACAC F: CGGCGGTGGAATGTAAGGAAGT R: GTGCAGGGTCCGAGGT	(Huang et al. 2012)
miR-let-7a	SL: GTCGTATCCAGTGCAGGGTCCGAGGTATTCGCACTGGATACGACAACTA F: GCCGCTGAGGTAGTAGGTTGTA R: GTGCAGGGTCCGAGGT	(Blödorn et al. 2023)

### Gene and microRNA expression analysis

Quantitative real-time PCR (qPCR) analyses were conducted using the GoTaq® qPCR Master Mix (Promega Corporation, USA) on a QuantStudio Real-Time PCR System (Applied Biosystems, USA), following the manufacturer’s recommended cycling parameters. Primer sets used for this study had been previously validated in *Oreochromis niloticus* (Huang et al. 2012). The target genes included key regulators of growth: growth hormone I (*gh1*), growth hormone receptor a (*ghra*), insulin-like growth factor I (*igf-1*), and insulin-like growth factor II (*igf-2*). For normalization of mRNA expression, β-actin (*actb*) was used as the reference gene, based on prior research (Yang et al. 2013).

MicroRNAs were selected from existing literature due to their known involvement in growth regulation. Putative interactions between selected miRNAs and target genes were predicted using the TargetScanFish database (https://www.targetscan.org/fish_62/), referencing the *Danio rerio* genome for alignment. These predictions were used solely to support biological plausibility and were not experimentally validated.

Primer sequences used for both mRNAs and miRNAs are listed in Tables 1 and 2. All qPCR reactions were carried out in duplicate. Relative gene expression levels were calculated using the 2^−ΔΔCT^ method, employing *actb* as the internal control for mRNAs and *miR-let-7* for miRNAs (Blödorn et al. 2023; Livak and Schmittgen 2001; Yang et al. 2013).

**Table 2:** List of primer sequences used for real-time quantitative PCR (q-PCR) analysis in this study

Gene name	Gene symbol	Primer sequences (5’ → 3’)	GenBank accession no	**Reference**
Growth Hormone 1	*gh1*	F: TCGGTTGTGTGTTTGGGCGTCTC R: GTGCAGGTGCGTGACTCTGTTGA	XM003442542	(Qiang et al. 2017)
Growth Hormone Receptor a	*ghra*	F: ATGGCTCTCTCGCCCTCCTCTAA R: ATGTCGTGTGTTCCCAGTCAGTGA	NM001279601	(Qiang et al. 2017)
Insulin Like Growth Factor 1	*igf-1*	F: TTGTCTGTGGAGAGCGAGGCTT R: CAGCTTTGGAAGCAGCACTCGT	NM001279503	(Qiang et al. 2017)
Insulin Like Growth Factor 2	*igf-2*	F: CCCCTGATCAGCCTTCCTA R: GACAAAGTTGTCCGTGGTGA	EU272150	(Wang et al. 2008)
β-actin	*actb*	F: TGGTGGGTATGGGTCAGAAAG R: CTGTTGGCTTTGGGGTTCA	XM003455949	(Yang et al. 2013)

### Statistical analysis

Normality and homoscedasticity of the data were assessed using the Shapiro–Wilk test and F test, respectively. Gene and miRNA expression levels were compared between control and cold-exposed groups using Student’s t-test, considering individual fish as independent biological replicates. Because no pooling was performed and each fish was treated as an experimental unit, more complex mixed or nested models were not applied. Statistical analyses were performed using Statistix version 10.0 software. Graphs were generated using GraphPad Prism 8.0. Data are presented as mean ± standard error of the mean (SEM). Statistical significance was set at *p* < 0.05, with additional significance thresholds indicated in the figures and Results section as follows: *p* < 0.01, *p* < 0.001, and *p* < 0.0001.

## Results

### Relative expression of growth-related genes

**Fig. 1:**
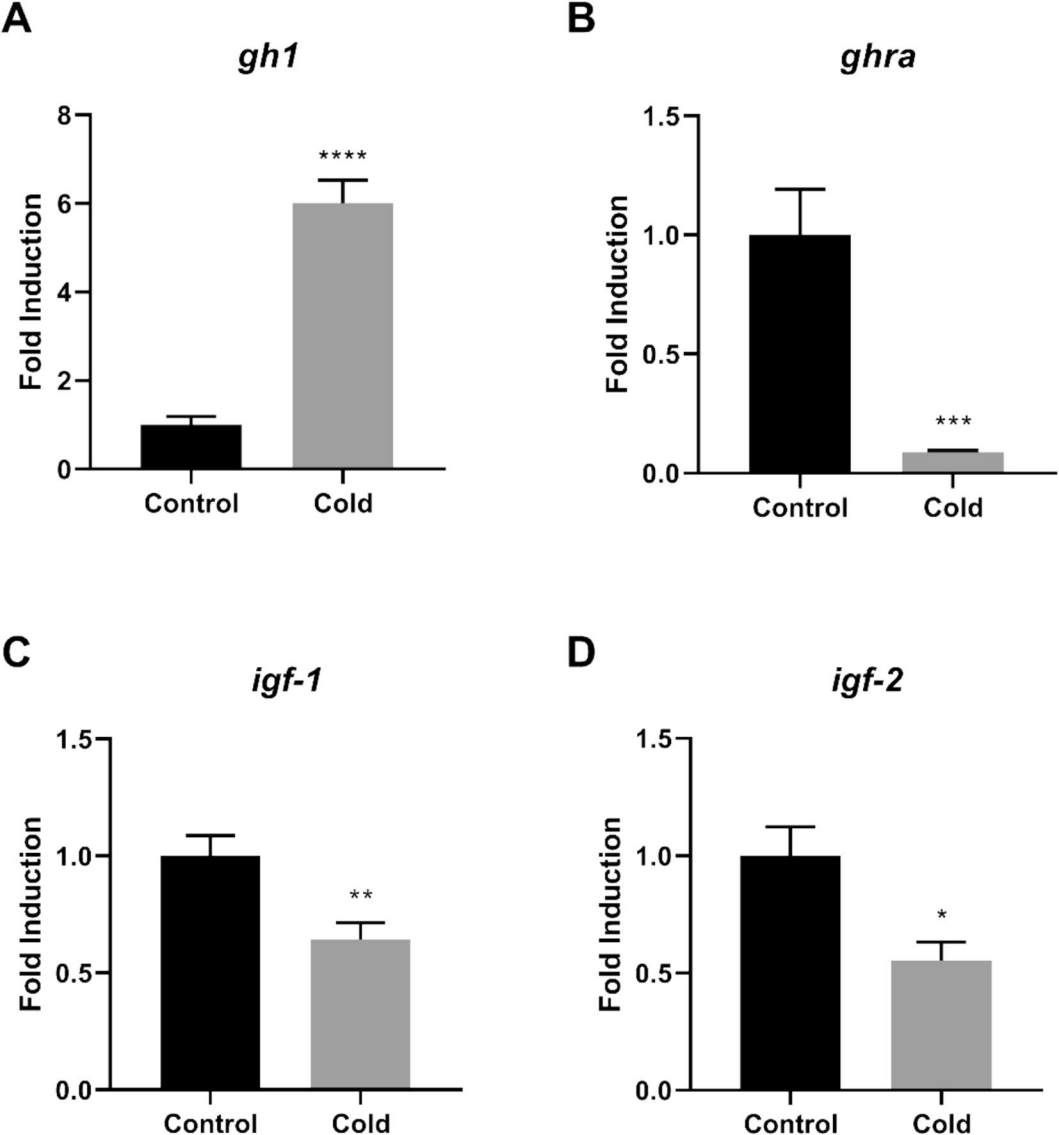
Relative gene expression in the brain of Nile tilapia (*Oreochromis niloticus*) from the control and cold-exposed groups. The mRNA expression levels of *gh1* (**A**), *ghra* (**B**), *igf-1* (**C**), and *igf-2* (**D**) were quantified by quantitative reverse transcription–polymerase chain reaction (RT-qPCR) and normalized to the *actb* reference gene. Data are presented as mean ± standard error of the mean (SEM). Asterisks indicate significant differences between experimental groups (Student’s t-test;* n* = 6 fish per group*;* * *p* < 0.05, ** *p* < 0.01, *** *p* < 0.001 and **** *p* < 0.0001)

**Fig. 2:**
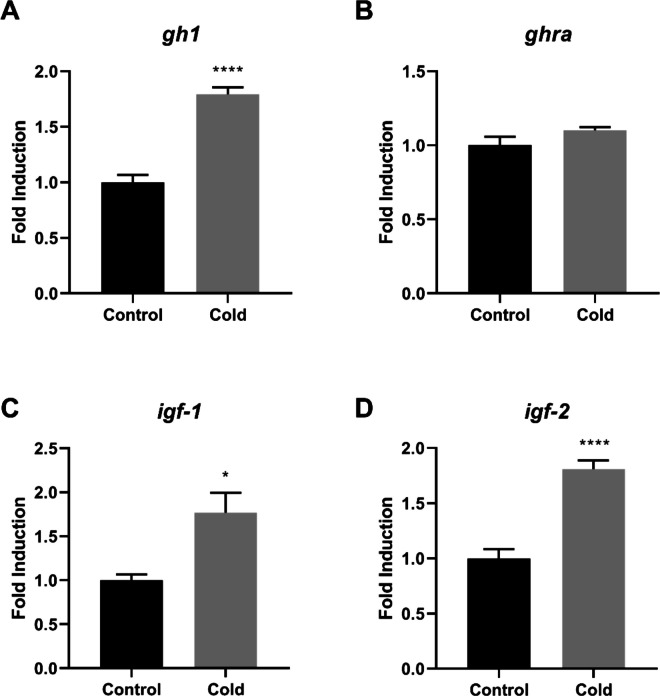
Relative gene expression in the liver of Nile tilapia (*Oreochromis niloticus*) from the control and cold-exposed groups. The mRNA expression levels of *gh1* (**A**), *ghra* (**B**), *igf-1* (**C**), and *igf-2* (**D**) were quantified by quantitative reverse transcription–polymerase chain reaction (RT-qPCR) and normalized to the *actb* reference gene. Data are presented as mean ± standard error of the mean (SEM). Asterisks indicate significant differences between experimental groups (Student’s t-test;* n* = 6 fish per group*;* * *p* < 0.05, ** *p* < 0.01, *** *p* < 0.001 and **** *p* < 0.0001)

**Fig. 3:**
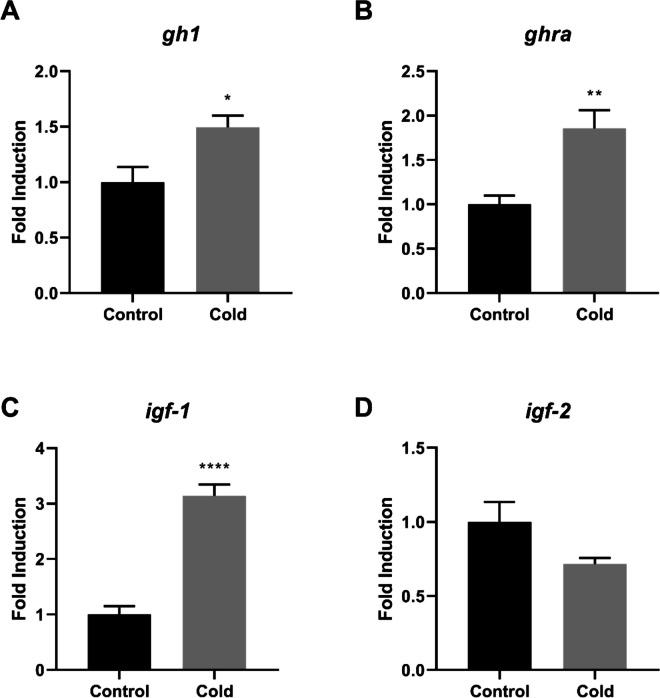
Relative gene expression in the hindgut of Nile tilapia (*Oreochromis niloticus*) from the control and cold-exposed groups. The mRNA expression levels of *gh1* (**A**), *ghra* (**B**), *igf-1* (**C**), and *igf-2* (**D**) were quantified by quantitative reverse transcription–polymerase chain reaction (RT-qPCR) and normalized to the *actb* reference gene. Data are presented as mean ± standard error of the mean (SEM). Asterisks indicate significant differences between experimental groups (Student’s t-test;* n* = 6 fish per group*;* * *p* < 0.05, ** *p* < 0.01, *** *p* < 0.001 and **** *p* < 0.0001)

The relative expression of growth-related genes was evaluated in the brain, liver, and hindgut of tilapia exposed to low temperatures, as shown in Figs. 1, 2, and 3, respectively. In the brain, *gh1* expression was significantly higher in the cold-exposed group compared with the control group (*p* < 0.0001) (Fig. 1A), whereas *ghra*, *igf-1*, and *igf-2* expression levels were significantly lower after 28 days of cold exposure (*p* < 0.05, *p* < 0.01, and *p* < 0.001, respectively) (Fig. 1B–D).

In the liver, *gh1, igf-1,* and *igf-2* expression levels were significantly elevated in cold-exposed fish compared with the control group (*p* < 0.05 and *p* < 0.0001; Fig. 2A, C, and D). No significant differences were detected in *ghra* expression between groups (Fig. 2B).

In the hindgut, cold exposure resulted in significantly higher expression of *gh1, ghra,* and *igf-1* (*p* < 0.05, *p* < 0.01, and *p* < 0.0001, respectively; Fig. 3A–C), whereas *igf-2* expression did not differ significantly between experimental groups (Fig. 3D).

### Relative expression of microRNAs

**Fig. 4:**
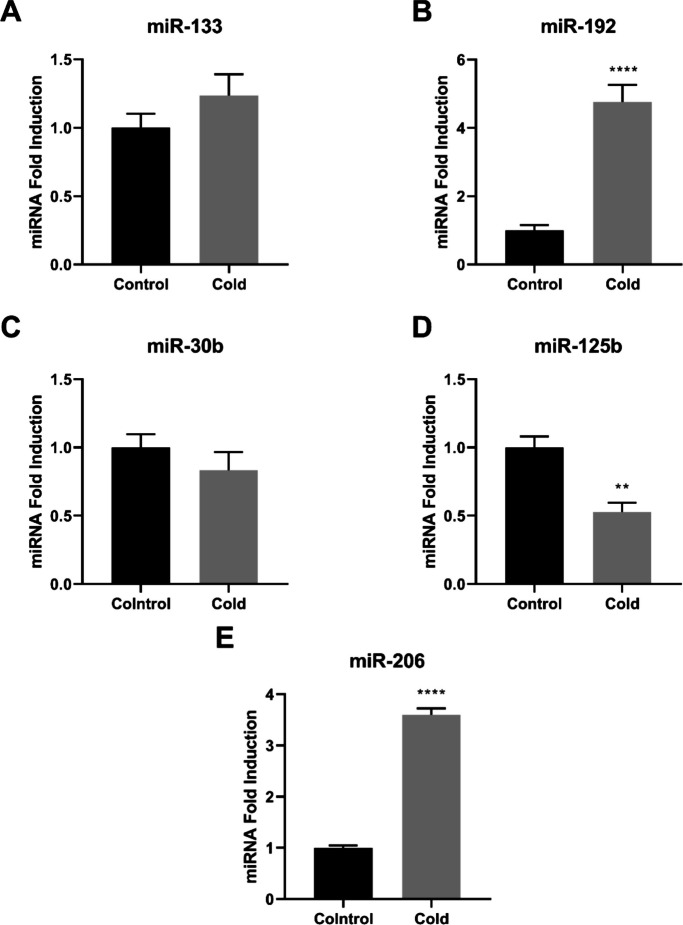
miRNA expression in the brain of Nile tilapias (*O. niloticus*) from the control group and from the cold group. The miRNA relative expression of miR-133 (**A**), miR-192 (**B**), miR-30b (**C**), miR-125b (**D**)*,* and miR-206 (**E**) were quantified by quantitative reverse transcription–polymerase chain reaction (RT-qPCR) and normalized using the miR-let-7 as reference. The values are expressed as mean ± standard error of the mean. Asterisk indicates significant differences between the experimental groups (Student’s t-test;* n* = 6 fish per group; ** *p* < 0.01 and **** *p* < 0.0001)

**Fig. 5:**
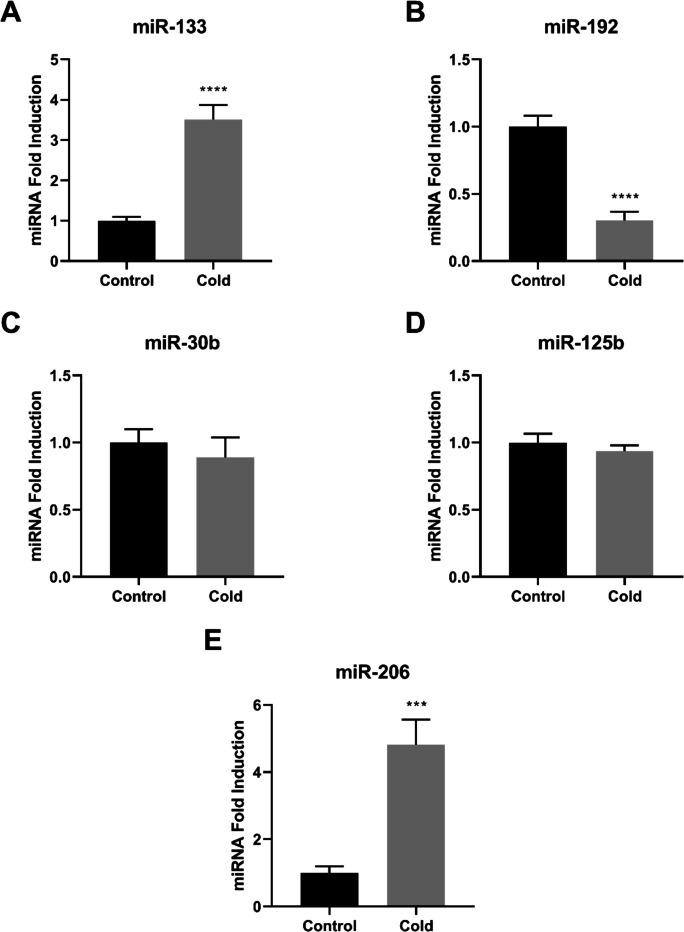
miRNA expression in the liver of Nile tilapias (*O. niloticus*) from the control group and from the cold group. The miRNA relative expression of miR-133 (**A**), miR-192 (**B**), miR-30b (**C**), miR-125b (**D**), and miR-206 (**E**) were quantified by quantitative reverse transcription–polymerase chain reaction (RT-qPCR) and normalized using the miR-let-7 as reference. The values are expressed as mean ± standard error of the mean. Asterisk indicates significant differences between the experimental groups (Student’s t-test;* n* = 6 fish per group; * *p* < 0.05, *** *p* < 0.001 and **** *p* < 0.0001)

**Fig. 6:**
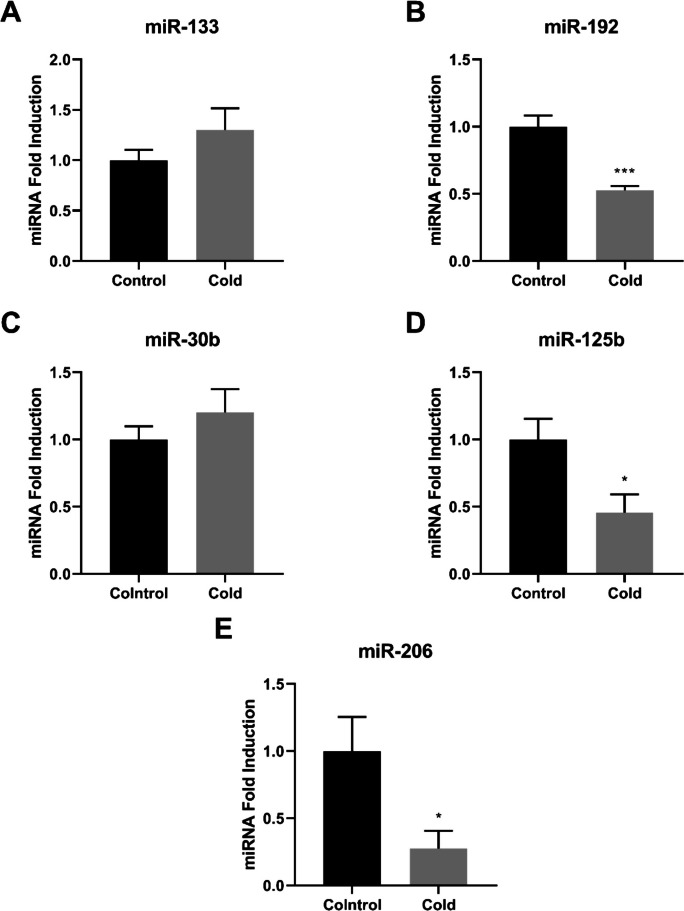
miRNA expression in the hindgut of Nile tilapias (*O. niloticus*) from the control group and from the cold group. The miRNA relative expression of miR-133 (**A**), miR-192 (**B**), miR-30b (**C**), miR-125b (**D**), and miR-206 (**E**) were quantified by quantitative reverse transcription–polymerase chain reaction (RT-qPCR) and normalized using the miR-let-7 as reference. The values are expressed as mean ± standard error of the mean. Asterisk indicates significant differences between the experimental groups (Student’s t-test;* n* = 6 fish per group; * *p* < 0.05 and *** *p* < 0.001)

MicroRNA expression analysis revealed distinct tissue-dependent responses to chronic low-temperature exposure (Figs. 4, 5, 6).

In the brain, cold-exposed fish exhibited significantly higher expression of miR-192 and miR-206 compared with controls (*p* < 0.0001; Fig. 4B and E). Conversely, miR-125b expression was significantly reduced under cold conditions (*p* < 0.01; Fig. 4D). No significant differences were observed for miR-133 or miR-30b (*p* > 0.05; Fig. 4A and C).

In the liver, expression levels of miR-133 and miR-206 were significantly increased in the cold-exposed group (*p* < 0.001 and *p* < 0.0001; Fig. 5A and E), whereas miR-192 expression was significantly reduced (*p* < 0.0001; Fig. 5B). No significant changes were detected for miR-30b or miR-125b (*p* > 0.05; Fig. 5C and D).

In the hindgut, cold exposure resulted in significantly lower expression of miR-192, miR-125b, and miR-206 compared with controls (*p* < 0.05 and *p* < 0.001; Fig. 6B, D, and E). Expression levels of miR-133 and miR-30b did not differ significantly between groups (*p* > 0.05; Fig. 6A and C).

## Discussion

Nile tilapia (*O. niloticus*) is one of the most widely cultured fish species worldwide, valued for its adaptability and rapid growth. However, its pronounced sensitivity to low temperatures represents a major challenge for aquaculture, particularly in subtropical and temperate regions. Understanding the molecular mechanisms underlying cold tolerance is therefore essential for improving resilience and ensuring sustainable production. The present study demonstrates that chronic cold exposure alters both endocrine growth signalling and miRNA-mediated regulation in a tissue-dependent manner, indicating that thermal adaptation in tilapia involves coordinated transcriptional and post-transcriptional responses rather than a generalized suppression of growth-related pathways.

The growth hormone/growth hormone receptor/insulin-like growth factor (GH/GHR/IGF) axis is a central regulator of somatic growth, nutrient allocation, and stress physiology in vertebrates (Triantaphyllopoulos et al. 2020). In fish, this axis is highly sensitive to environmental variables, including temperature (Chang and Wong 2009; Donaldson et al. 2008; Mininni et al. 2014). In the present study, chronic cold exposure increased *gh1* expression in all tissues analyzed, suggesting activation of a broad compensatory response to prolonged thermal challenge. Although GH is classically synthesized in the pituitary, extra-pituitary expression has been described in teleosts and may reflect local regulatory mechanisms related to tissue maintenance, metabolic adjustment, or stress adaptation (Petro-Sakuma et al. 2020).

The target genes evaluated here showed markedly distinct tissue-specific patterns, which likely reflect the different physiological roles of the brain, liver, and hindgut during chronic cold exposure. In the liver, the upregulation of *gh1*, *igf-1*, and *igf-2* suggests activation of pathways associated with endocrine and metabolic compensation. Because the liver is the main organ responsible for integrating nutrient availability with systemic growth signalling, this response may represent an attempt to preserve anabolic and metabolic balance under conditions of reduced temperature and impaired feed intake. Similarly, the increase in *gh1*, *ghra*, and *igf-1* expression in the hindgut may reflect local adaptive responses aimed at maintaining intestinal integrity, nutrient transport capacity, or tissue homeostasis during cold stress. In contrast, the brain displayed a different pattern, characterized by increased *gh1* but reduced *ghra*, *igf-1*, and *igf-2* expression. This profile suggests that neural tissue may respond to cold exposure through tighter regulation of growth-related signalling, possibly prioritizing energy conservation and central homeostatic control rather than local growth-promoting activity.

These observations reinforce that increased transcription of growth-related genes under cold exposure should not be interpreted as evidence of enhanced somatic growth. Instead, the upregulation observed in metabolically relevant tissues such as liver and hindgut likely reflects compensatory molecular responses to chronic cold stress. This interpretation is consistent with previous findings from our research group showing that prolonged exposure to low temperatures suppresses feeding and compromises physiological performance in Nile tilapia (Dellagostin et al. 2022). Thus, the activation of components of the GH/GHR/IGF axis may represent an attempt to maintain metabolic equilibrium despite environmental conditions that are unfavorable for actual growth.

In addition to transcriptional regulation, post-transcriptional control mediated by miRNAs appears to be an important component of the cold response observed here. The miRNA profiles were clearly tissue-dependent, indicating that chronic cold stress does not induce a single regulatory signature across the organism (Lin et al. 2025). Instead, each tissue appears to engage distinct miRNA-mediated mechanisms according to its physiological role. This point is especially relevant because the biological significance of miRNAs is not simply whether they are up- or downregulated, but how their expression patterns relate to the behavior of putative target genes within a given tissue context.

Among the miRNAs evaluated, miR-192 showed one of the most informative patterns. In the brain, miR-192 was upregulated while *igf-1* and *igf-2* were reduced, suggesting a possible inverse relationship between this miRNA and growth-related signalling in neural tissue. MiR-192 has been associated with insulin-related and metabolic pathways and has been linked to components of the IGF-binding protein network (Li et al. 2021; Van Gelderen et al. 2025). Although no direct interaction was validated in the present study, this expression pattern is compatible with the hypothesis that miR-192 contributes to the suppression or fine control of growth-related signalling in the brain during chronic cold stress. Such regulation may be relevant in a tissue where energy-demanding anabolic processes are likely deprioritized in favor of maintaining essential neural functions under adverse environmental conditions.

A similarly important result was the co-upregulation of miR-206 and *igf-1* in the liver. MiR-206 has previously been validated as a post-transcriptional regulator of *igf-1* in Nile tilapia (Yan et al. 2012), and in many contexts its induction is associated with attenuation of IGF signalling. In this study, however, both molecules increased in parallel under cold exposure. This apparent discrepancy suggests that the relationship between miR-206 and *igf-1* may be more complex than a simple inhibitory interaction. One possible explanation is that miR-206 acts as a fine-tuning regulator, modulating *igf-1* expression without fully suppressing it. Another possibility is that strong transcriptional stimulation of *igf-1* in the liver under chronic cold conditions may override or partially counterbalance miRNA-mediated repression (Lin et al. 2013). Either interpretation supports the idea that miRNA–mRNA relationships during long-term environmental stress are dynamic, context-dependent, and strongly influenced by tissue-specific physiological demands.

MiR-133 also deserves consideration in relation to the target genes analyzed. Its upregulation in the liver, together with the induction of components of the GH/GHR/IGF axis, may indicate that this miRNA participates in hepatic metabolic remodeling rather than in a direct inhibitory response (Mishima et al. 2009). Because miR-133 has been associated with muscle development and IGF-related signalling in previous studies, its altered expression in liver under cold exposure may reflect broader systemic adaptation involving energy redistribution and growth regulation. In contrast, its stable expression in brain and hindgut suggests that its contribution to chronic cold adaptation is tissue-restricted rather than generalized.

MiR-125b exhibited another relevant pattern, being downregulated in both brain and hindgut. This result may indicate reduced repression of pathways associated with survival, metabolism, or stress adaptation in these tissues (Valmiki et al. 2020; Wang et al. 2019). Because miR-125b has been implicated in the regulation of PI3K/Akt-related signalling and immune responses in other systems (Fu et al. 2020; Liang et al. 2025; Lee et al. 2016), its downregulation under chronic cold exposure may be associated with the release of compensatory pathways required for tissue maintenance under adverse thermal conditions. Although this interpretation remains speculative, the combined behavior of miR-125b and the growth-related genes supports the idea that cold adaptation involves extensive reorganization of regulatory networks rather than isolated expression changes.

In contrast to the other candidate miRNAs, miR-30b remained stable in all tissues analyzed. This finding is noteworthy because previous studies have proposed miR-30b as a potential biomarker of acute thermal stress in teleosts (Blödorn et al. 2021). Its lack of differential expression in the present study may indicate that miR-30b is more closely associated with early or transient responses to cold exposure than with long-term adaptation. This distinction between acute and chronic regulatory responses is important, as biomarkers identified under short-term stress conditions may not necessarily remain informative during prolonged exposure.

Overall, the findings of this study indicate that chronic cold exposure in Nile tilapia induces a coordinated but non-uniform regulatory response involving both growth-related genes and miRNAs. The liver and hindgut appear to mount compensatory transcriptional responses that may help sustain metabolic and physiological stability, whereas the brain exhibits a more restrictive growth-related profile, possibly associated with central energy conservation. At the same time, tissue-specific miRNA modulation suggests that post-transcriptional regulation contributes substantially to shaping these responses and may help explain why activation of growth-related genes does not necessarily translate into enhanced growth performance under cold conditions.

Some limitations of this study should be acknowledged. The conclusions are based on correlative analyses of mRNA and miRNA expression and therefore do not demonstrate direct miRNA–target interactions. Functional validation approaches, such as luciferase reporter assays, miRNA gain- and loss-of-function experiments, and protein-level analyses, will be necessary to confirm the regulatory relationships proposed here. Even so, the tissue-specific patterns identified in this study provide a valuable framework for understanding chronic cold adaptation in Nile tilapia and may support future efforts to identify molecular markers of cold tolerance for selective breeding programs.

## Conclusion

In conclusion, this study provides integrative insights into the molecular responses associated with chronic cold exposure in Nile tilapia, revealing coordinated transcriptional and post-transcriptional regulation across metabolically relevant tissues. The results highlight the involvement of the growth hormone/insulin-like growth factor (GH/IGF) axis, specifically *gh*, *ghr*, *igf-1*, and *igf-2*, in mediating tissue-specific adjustments to prolonged low-temperature conditions. Marked transcriptional responses were observed in the liver and hindgut, whereas the brain exhibited a more heterogeneous and tightly regulated expression profile, suggesting distinct physiological roles of each tissue during cold adaptation.

The differential regulation of miRNAs, including miR-192, miR-206, miR-133, miR-125b, and miR-30b, further underscores the complexity of post-transcriptional control under thermal stress. The tissue-dependent expression patterns observed here indicate that miRNAs may contribute to fine-tuning growth-related and metabolic pathways in response to chronic cold exposure, potentially balancing energy conservation with the maintenance of essential cellular functions. Notably, the concomitant regulation of miRNAs and growth-related genes suggests coordinated, but context-dependent, regulatory interactions rather than uniform inhibitory or activating effects.

Importantly, the conclusions of this study are based on correlative analyses of mRNA and miRNA expression and do not provide direct evidence of functional miRNA–target interactions or downstream protein-level effects. Moreover, growth performance and physiological parameters were not assessed, limiting direct inference regarding phenotypic outcomes of the observed molecular responses. Future studies integrating functional validation approaches, such as luciferase reporter assays, miRNA modulation experiments, and proteomic analyses, will be necessary to confirm the regulatory mechanisms proposed and to determine their physiological relevance.

Despite these limitations, the tissue-specific regulatory patterns identified here provide a valuable framework for understanding molecular adaptation to chronic cold stress in Nile tilapia. These findings may inform future research aimed at identifying molecular markers associated with cold tolerance, supporting long-term strategies for improving resilience in tilapia aquaculture under fluctuating temperature regimes.

## Supplementary Information

Below is the link to the electronic supplementary material.

Supplementary file1 (XLSX 18 KB)

## Data Availability

The data supporting the findings in this study is available in the Supplementary Material section.

## References

[CR1] Abd El-Hack ME, El-Saadony MT, Nader MM, Salem HM, El-Tahan AM, Soliman SM, Khafaga AF (2022) Effect of environmental factors on growth performance of Nile tilapia (*Oreochromis niloticus*). Int J Biometeorol 66:2183–2194. 10.1007/s00484-022-02347-6 36044083 10.1007/s00484-022-02347-6 PMC9640449

[CR2] Abram Q, Dixon B, Katzenback B (2017) Impacts of low temperature on the teleost immune system. Biology 6:39. 10.3390/biology6040039 29165340 10.3390/biology6040039 PMC5745444

[CR3] Bertucci JI, Blanco AM, Sundarrajan L, Rajeswari JJ, Velasco C, Unniappan S (2019) Nutrient regulation of endocrine factors influencing feeding and growth in fish. Front Endocrinol 10:83. 10.3389/fendo.2019.00083 10.3389/fendo.2019.00083 PMC6403160 30873115

[CR4] Bhattacharya M, Sharma AR, Sharma G, Patra BC, Nam J-S, Chakraborty C, Lee S-S (2017) The crucial role and regulations of miRNAs in zebrafish development. Protoplasma 254:17–31. 10.1007/s00709-015-0931-1 26820151 10.1007/s00709-015-0931-1

[CR5] Biga PR, Meyer J (2009) Growth hormone differentially regulates growth and growth-related gene expression in closely related fish species. Comp Biochem Physiol A Mol Integr Physiol 154:465–473. 10.1016/j.cbpa.2009.07.023 19654052 10.1016/j.cbpa.2009.07.023

[CR6] Bizuayehu TT, Babiak I (2014) MicroRNA in teleost fish. Genome Biol Evol 6:1911–1937. 10.1093/gbe/evu151 25053657 10.1093/gbe/evu151 PMC4159003

[CR7] Blödorn EB, Domingues WB, Nunes LS, Komninou ER, Pinhal D, Campos VF (2021) MicroRNA roles and their potential use as selection tool to cold tolerance of domesticated teleostean species: a systematic review. Aquaculture 540:736747. 10.1016/j.aquaculture.2021.736747

[CR8] Blödorn EB, Domingues WB, Martins AWS, Dellagostin EN, Komninou ER, Remião MH, Silveira TLR, Collares GL, Giongo JL, Vaucher RA, Campos VF (2023) MicroRNA qPCR normalization in Nile tilapia (*Oreochromis niloticus*): effects of acute cold stress on potential reference targets. Fish Physiol Biochem 49:409–423. 10.1007/s10695-023-01190-9 37074474 10.1007/s10695-023-01190-9

[CR9] Blödorn EB, Martins AWS, Dellagostin EN, Nunes LS, Da Conceição RCS, Pagano AD, Gonçalves NM, Dos Reis LFV, Nascimento MC, Quispe DKB, Fróes CN, Tavares RA, Giongo JL, Vaucher RA, Robaldo RB, Domingues WB, Collares GL, Pinhal D, Campos VF (2024) Toward new biomarkers of cold tolerance: microRNAs regulating cold adaptation in fish are differentially expressed in cold-tolerant and cold-sensitive Nile tilapia (*Oreochromis niloticus*). Aquaculture 589:740942. 10.1016/j.aquaculture.2024.740942

[CR10] Canosa LF, Bertucci JI (2023) The effect of environmental stressors on growth in fish and its endocrine control. Front Endocrinol 14:1109461. 10.3389/fendo.2023.1109461 10.3389/fendo.2023.1109461 PMC10098185 37065755

[CR11] Cao Q, Zhang H, Li T, He L, Zong J, Shan H, Huang L, Zhang Y, Liu H, Jiang J (2023) Profiling miRNAs of teleost fish in responses to environmental stress: a review. Biology 12:388. 10.3390/biology12030388 36979079 10.3390/biology12030388 PMC10045198

[CR12] Chang JP, Wong AOL (2009) Growth hormone regulation in fish, In: Fish Physiology, 4. Elsevier, pp. 151–195. 10.1016/S1546-5098(09)28004-6

[CR13] Chen C, Ridzon DA, Broomer AJ, Zhou Z, Lee DH, Nguyen JT, Barbisin M, Xu NL, Mahuvakar VR, Andersen MR, Lao KQ, Livak KJ, Guegler KJ (2005) Real-time quantification of microRNAs by stem-loop RT-PCR. Nucleic Acids Res 33(20):e179. 10.1093/nar/gni178 16314309 10.1093/nar/gni178 PMC1292995

[CR14] Dellagostin EN, Martins AWS, Blödorn EB, Silveira RTL, Komninou ER, Varela Junior AS, Corcini CD, Nunes LS, Remião MH, Collares GL, Domingues WB, Giongo JL, Vaucher RA, Campos VF (2022) Chronic cold exposure modulates genes related to feeding and immune system in Nile tilapia (*Oreochromis niloticus*). Fish Shellfish Immunol 128:269–278. 10.1016/j.fsi.2022.07.075 35952998 10.1016/j.fsi.2022.07.075

[CR15] Donaldson MR, Cooke SJ, Patterson DA, Macdonald JS (2008) Cold shock and fish. J Fish Biol 73:1491–1530. 10.1111/j.1095-8649.2008.02061.x

[CR16] Escobar-Aguirre S, Felip A, Mazón MJ, Ballester-Lozano G, Pérez-Sánchez J, Björsson BT, Zanuy S, Carrillo M (2020) Long-term feeding of a maintenance ration affects the release of Igf-1 and leptin, and delays maturation in a male teleost fish, *Dicentrarchus labrax* L. Aquaculture 527:735467. 10.1016/j.aquaculture.2020.735467

[CR17] FAO (2024) The state of world fisheries and aquaculture 2024. FAO, Rome. 10.4060/cd0683en

[CR18] Fu K, Zhang L, Liu R, Shi Q, Li X, Wang M (2020) MiR-125 inhibited cervical cancer progression by regulating VEGF and PI3K/AKT signaling pathway. World J Surg Oncol 18:115. 10.1186/s12957-020-01881-0 32473637 10.1186/s12957-020-01881-0 PMC7261381

[CR19] Herkenhoff ME, Oliveira AC, Nachtigall PG, Costa JM, Campos VF, Hilsdorf AWS, Pinhal D (2018) Fishing into the MicroRNA transcriptome. Front Genet 9:88. 10.3389/fgene.2018.00088 29616080 10.3389/fgene.2018.00088 PMC5868305

[CR20] Huang CW, Li YH, Hu SY, Chi JR, Lin GH, Lin CC, Gong HY, Chen JY, Chen RH, Chang SJ, Liu FG, Wu JL (2012) Differential expression patterns of growth-related microRNAs in the skeletal muscle of Nile tilapia (*Oreochromis niloticus*). J Anim Sci 90:4266–4279. 10.2527/jas.2012-5142 22745188 10.2527/jas.2012-5142

[CR21] Lee H-M, Kim TS, Jo E-K (2016) MiR-146 and miR-125 in the regulation of innate immunity and inflammation. BMB Rep 49:311–318. 10.5483/BMBRep.2016.49.6.056 26996343 10.5483/BMBRep.2016.49.6.056 PMC5070718

[CR22] Li L, Huang Y, Zhang Z (2021) Expression profile of miRNAs involved in the hepatoprotective effects of curcumin against oxidative stress in Nile tilapia. Aquat Toxicol 237:105896. 10.1016/j.aquatox.2021.105896 34174576 10.1016/j.aquatox.2021.105896

[CR23] Liang C, Zhuoer Z, Yuan L, Bing Z (2025) Triptolide inhibits migration and viability, and promotes apoptosis by targeting the PI3K/Akt signaling pathway via upregulation of miR-125a-5p in SW1353 human chondrosarcoma cells. Mol Med Rep 31:1–12. 10.3892/mmr.2025.13514 40211712 10.3892/mmr.2025.13514

[CR24] Lin T, Meegaskumbura M (2025) Fish MicroRNA responses to thermal stress: insights and implications for aquaculture and conservation amid global warming. Animals 15:624. 10.3390/ani15050624 40075907 10.3390/ani15050624 PMC11898199

[CR25] Lin C-Y, Lee H-C, Fu C-Y, Ding Y-Y, Chen J-S, Lee M-H, Huang W-J, Tsai H-J (2013) miR-1 and miR-206 target different genes to have opposing roles during angiogenesis in zebrafish embryos. Nat Commun 4:2829. 10.1038/ncomms3829 24264597 10.1038/ncomms3829

[CR26] Livak KJ, Schmittgen TD (2001) Analysis of relative gene expression data using real-time quantitative PCR and the 2(-Delta Delta C(T)) method. Methods (San Diego, Calif) 25(4):402–408. 10.1006/meth.2001.1262 11846609 10.1006/meth.2001.1262

[CR27] Melo RMC, Ribeiro YM, Luz RK, Bazzoli N, Rizzo E (2016) Influence of low temperature on structure and dynamics of spermatogenesis during culture of *Oreochromis niloticus*. Anim Reprod Sci 172:148–156. 10.1016/j.anireprosci.2016.07.013 27477114 10.1016/j.anireprosci.2016.07.013

[CR28] Mininni AN, Milan M, Ferraresso S, Petochi T, Di Marco P, Marino G, Livi S, Romualdi C, Bargelloni L, Patarnello T (2014) Liver transcriptome analysis in gilthead sea bream upon exposure to low temperature. BMC Genomics 15:765. 10.1186/1471-2164-15-765 25194679 10.1186/1471-2164-15-765 PMC4167152

[CR29] Mishima Y, Abreu-Goodger C, Staton AA, Stahlhut C, Shou C, Cheng C, Gerstein M, Enright AJ, Giraldez AJ (2009) Zebrafish miR-1 and miR-133 shape muscle gene expression and regulate sarcomeric actin organization. Genes Dev 23:619–632. 10.1101/gad.1760209 19240126 10.1101/gad.1760209 PMC2658521

[CR30] Pedrini B, Urbano C, Dellova D, Souza F, Oliveira G, Dias I, Fabris I, Estrela J, Real JV, Albuquerque L, Amaral L, Tabatiano M, Iglesias R, Cândido R, Passerini V, Medeiros F (2024) Anuário Brasileiro da Piscicultura

[CR31] Petro-Sakuma C, Celino-Brady FT, Breves JP, Seale AP (2020) Growth hormone regulates intestinal gene expression of nutrient transporters in tilapia (*Oreochromis mossambicus*). Gen Comp Endocrinol 292:113464. 10.1016/j.ygcen.2020.113464 32171745 10.1016/j.ygcen.2020.113464 PMC7253219

[CR32] Qiang J, Bao JW, Li HX, Chen DJ, He J, Tao YF, Xu P (2017) miR-1338-5p modulates growth hormone secretion and glucose utilization by regulating ghitm in Genetically Improved Farmed Tilapia (GIFT, *Oreochromis niloticus*). Front Physiol 8:998. 10.3389/fphys.2017.00998 29270127 10.3389/fphys.2017.00998 PMC5723647

[CR33] Qiang J, Cui YT, Tao FY, Bao WJ, He J, Li XH, Xu P, Sun LY (2018) Physiological response and microRNA expression profiles in head kidney of genetically improved farmed tilapia (GIFT, *Oreochromis niloticus*) exposed to acute cold stress. Sci Rep 8:172. 10.1038/s41598-017-18512-6 29317697 10.1038/s41598-017-18512-6 PMC5760732

[CR34] Silva AdosS, Barros LSS (2020) Food safety and fish farming: serious issues for Brazil. FNS 11:123–152. 10.4236/fns.2020.112011

[CR35] Triantaphyllopoulos KA, Cartas D, Miliou H (2020) Factors influencing *GH* and *IGF-I* gene expression on growth in teleost fish: how can aquaculture industry benefit? Rev Aquacult 12:1637–1662. 10.1111/raq.12402

[CR36] Valenti WC, Barros HP, Moraes-Valenti P, Bueno GW, Cavalli RO (2021) Aquaculture in Brazil: past, present and future. Aquac Rep 19:100611. 10.1016/j.aqrep.2021.100611

[CR37] Valmiki S, Ahuja V, Puri N, Paul J (2020) MiR-125b and miR-223 contribute to inflammation by targeting the key molecules of NFκB pathway. Front Med 6:313. 10.3389/fmed.2019.00313 10.3389/fmed.2019.00313 PMC6990118 32039213

[CR38] Van Gelderen TA, Debnath P, Joly S, Bertomeu E, Duncan N, Furones D, Ribas L (2025) Gonadal miRNomes and transcriptomes in infected fish reveal sexually dimorphic patterns of the immune response. Funct Integr Genomics 25:29. 10.1007/s10142-025-01537-w 39883212 10.1007/s10142-025-01537-w PMC11782434

[CR39] Wang D-S, Jiao B, Hu C, Huang X, Liu Z, Cheng CHK (2008) Discovery of a gonad-specific IGF subtype in teleost. Biochem Biophys Res Commun 367:336–341. 10.1016/j.bbrc.2007.12.136 18166148 10.1016/j.bbrc.2007.12.136

[CR40] Wang JK, Wang Z, Li G (2019) MicroRNA-125 in immunity and cancer. Cancer Lett 454:134–145. 10.1016/j.canlet.2019.04.015 30981762 10.1016/j.canlet.2019.04.015

[CR41] Yan B, Zhao L, Guo J, Zhao J (2012) miR-206 regulates the growth of the teleost tilapia ( *Oreochromis niloticus* ) through the modulation of IGF-1 gene expression. J Exp Biol jeb.079590. 10.1242/jeb.079590 10.1242/jeb.079590 23197102

[CR42] Yang CG, Wang XL, Tian J, Liu W, Wu F, Jiang M, Wen H (2013) Evaluation of reference genes for quantitative real-time RT-PCR analysis of gene expression in Nile tilapia (*Oreochromis niloticus*). Gene 527:183–192. 10.1016/j.gene.2013.06.013 23792389 10.1016/j.gene.2013.06.013

[CR43] Yu Z, Zhang M, Luo B, Jing H, Yu Y, Wang S, Luo S (2020) Lrp4 in hippocampal astrocytes serves as a negative feedback factor in seizures. Cell Biosci 10:135. 10.1186/s13578-020-00498-w 33292473 10.1186/s13578-020-00498-w PMC7684739

[CR44] Zhou T, Gui L, Liu M, Li W, Hu P, Duarte DFC, Niu H, Chen L (2019) Transcriptomic responses to low temperature stress in the Nile tilapia, *Oreochromis niloticus*. Fish Shellfish Immunol 84:1145–1156. 10.1016/j.fsi.2018.10.023 30408600 10.1016/j.fsi.2018.10.023

